# Immunohistochemial study on the expression of von Willebrand factor (vWF) after onlay autogenous iliac grafts for lateral alveolar ridge augmentation

**DOI:** 10.1186/1746-160X-9-40

**Published:** 2013-12-11

**Authors:** Steffen Koerdt, Joerg Siebers, Wilhelm Bloch, Oliver Ristow, Alexander C Kuebler, Tobias Reuther

**Affiliations:** 1 Department of Oral and Maxillofacial Plastic Surgery, University of Wuerzburg, Pleicherwall 2, Wuerzburg D-97070, Germany; 2 Department of Molecular and Cellular Sport Medicine, German Sport University, Am Sportpark Muengersdorf 6, Cologne D-50933, Germany; 3 Medicine & Aesthetics, Clinic for Oral, Maxillofacial and Plastical Surgery, Lenbachplatz 2a, Munich D-80333, Germany

**Keywords:** GBR, Collagen membrane, Revascularization, Connective tissue, Bone graft, vWF

## Abstract

**Introduction:**

The main problems of autogenous bone transplants are their unpredictable atrophy and their loss of structure. One key factor lies in the poor revascularization of simple onlay grafts. The the aim of this study was to evaluate the revascularization processes in autogenous bone grafts from the iliac crest to the alveolar ridge.

**Methods:**

In a sheep model, autogenous bone grafts were harvested from the iliac crest. A combination of a resorbable collagen membrane (CM) and deproteinized bovine bone material (DBBM) was used to modify the bone graft (experiment 2). This was compared with a simple onlay bone graft (control group, experiment 1). The amount of vessels in bone and connective tissue (CT), and the amount of CT were analyzed. The expression of von Willebrand factor (vWF) was compared between the two experimental groups using immunohistochemical analysis.

**Results:**

The ratio of the amount of vessels in bone and CT changed over time, and more vessels could be detected in bone at 12–16 weeks of graft healing. The number of vessels were significantly higher in experiment 2 than in experiment 1. More CT was found in experiment 1, whereas the amount of CT in both experiments decreased over time.

**Conclusion:**

This study shows a more intensive and extensive revascularization in experiment 2, as significantly more vessels were detected. The decreased amount of CT in experiment 2 clarifies its clinical superiority.

## Introduction

Bone grafting is among the most frequently performed procedures with in oral surgery. Contiguous to their use in reconstruction of defects after tumor, trauma, or infections, bone transplants are also used for correcting syndromic defects and to create an adequate bone volume before the placement of dental implants. The replacement of bone as a support of the surrounding soft tissue is essential to ensure the functional and aesthetic rehabilitation of the patient. The gold standard is the transplantation of autogenous bone, which has the ability to maintain its osteogenic potential [1,2]. However, the main problems of autogenous bone transplants are their unpredictable atrophy and their loss of structure [3,4]. A decrease in the volume of nonfixed grafts of up to 50%-70% has been reported to occur during the first year after transplantation [5,6]. The size of the defect is a predetermined factor essential for the successful incorporation of a bone transplant. Adequate stabilization of the defect and the quality of the transplant as well as of the host site are considered key factors for a successful transplant healing. The quality of the host site can either be influenced by using anti-inflammatory medication or surgically through the concept of guided tissue regeneration (GTR), the use of bone substitution material, rigid fixation of the graft and inhibition of osteoclastic activities [5-14].

The concept of GTR was developed to prevent undesired cells from migrating into a defect using different kinds of barrier membranes. On the other hand, this allows certain desired cells to proliferate in the wound [15]. This concept was later employed in bone regeneration (guided bone regeneration, GBR) [16-20]. However, in clinical use, GBR does not always result in a predictable bone fill of the defect [17,20]. In experimental studies as well as in clinical use, a successful combination of onlay bone grafts with deproteinized bovine bone material (DBBM) has been reported [5,8]. Previous studies also showed the superiority of a combination of DBBM and GBR with the use of collagen membrane (CM). This current study adopts the results of Adeyemo et al. and attempts to put an emphasis on the revascularization procedures within the graft in the same experimental setting [3,4,21]. DBBMs in general, are considered to be biocompatible and osteoconductive; however, evidence about their biodegradability remains inconclusive [22,23].

One crucial factor for the successful incorporation of a transplant is its revascularization, as nutrients, gas, and undifferentiated mesenchymal cells are transported into the defect and bone regeneration through newly formed vessels is promoted [24,25]. Several studies described a close relation between new bone formation and revascularization [26,27]. Schmid et al. described, in a rabbit animal model, the close relation between the use of GBR and *de novo* extraskeletal bone formation when considering the effects of angiogenesis [25].

Only few reports on revascularization in bone grafts with and without GBR and DBBM are found in the literature. The aim of this study was to evaluate the different aspects of revascularization within the host site and the graft in a sheep model.

## Material and methods

Twelve adult female sheep were used in this study (mean weight ± standard deviation [SD], 73.6 ± 8.6 kg; range. 63–90 kg). The medical ethics committee of the University of Cologne, Germany, and local authorities approved the research reported in this article (institutional review board registration no 50.203.2 K43, 36/01). Depending on the time of euthanasia, the animals were randomized into four groups of three animals each.

### Anesthesia

After general anesthesia induction with 2% intravenous propofol, each animal was intubated. Anesthesia was maintained during the surgical procedure with (1) isoflurane, (2) oxygen, (3) propofol (2%), and (4) midazolam. In all animals, a perioperative antibiotic prophylaxis with penicillin-dihydrostreptomycin (aniMedica, Germany) was used for at least 3 days postoperatively. The animals also received analgesic treatment (Rimadyl; Pfizer, Germany) for 3 days after surgery, for pain management. At the end of surgery, anesthesia was terminated by the gradual wearing off of the effect of 2% propofol.

### Harvesting and transplantation of Iliac bone graft

**Figure 1:**
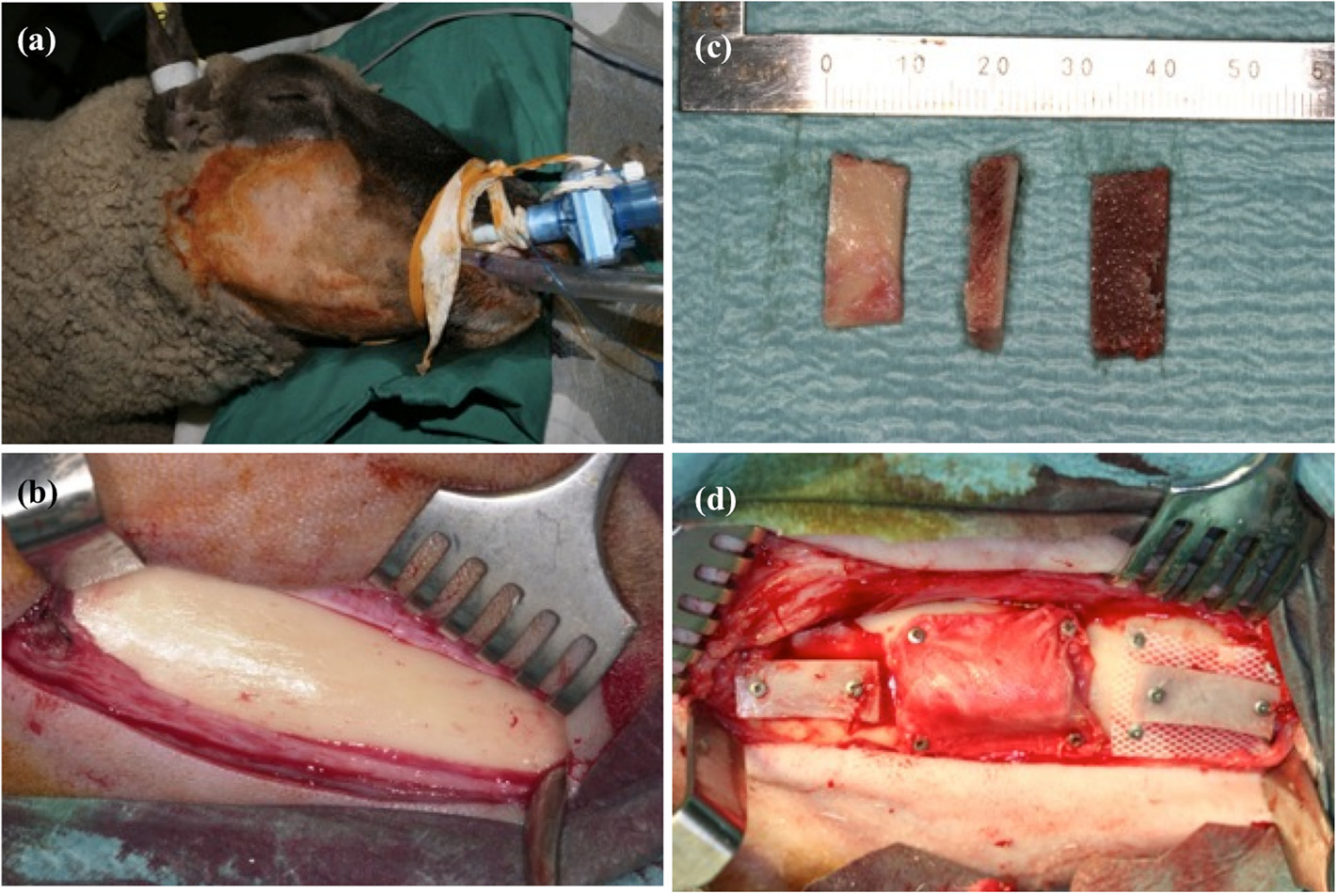
**Intraoperative situs: (a) Sheep were intubated and shaved preoperatively. (b)** Exposed lateral surface of the mandible before transplantation. **(c)** Monocortical bone grafts. **(d)** From left to right: experiment 1, experiment 2, and negative control with a non-porous silicone membrane.

All experimental procedures were performed by one of the investigators following a standardized protocol. Under general anesthesia, for all experiments performed, a bicortical bone graft (2.0 × 2.0 × 1.5 cm) was harvested from the iliac bone of each sheep. The harvested corticocancellous graft was divided into two equal sizes (1.0 × 2.0 × 1.5 cm), followed by splitting into two mono-cortical grafts each (1.0 × 2.0 × 0.75 cm, Figure 1). For all experiments the lateral surface of the mandible was carefully exposed through an extraoral surgical approach without perforation to the oral cavity in order to avoid excessive damage to the internal structures of the mandible (Figure 1). Each sheep received the experimental grafts on the lateral surface of the mandible (Figure 1). Surgical wounds were then closed in layers with interrupted resorbable sutures (Vicryl® 2.0; Ethicon, Norderstedt, Germany). All procedures performed in a strictly aseptic environment.

### Experiment 1

The cancellous bone portion of the graft was placed tightly against the mandibular cortical bone and fixed with two titanium screws. The screws were inserted into the graft until the undersurface of the screw heads came into contact with the outer surface of the graft.

### Experiment 2

The cancellous bone portion of the graft was placed tightly against the mandibular cortical bone and fixed with two titanium screws. In addition, Bio-Oss® spongiosa granules (0.25 - 1.0 mm) (Geistlich Pharma AG, Switzerland), moistened with blood, were applied around the graft and contoured well. The recipient site and the graft covered with Bio-Oss® were then covered with a Bio-Gide® CM (Geistlich Pharma AG, Switzerland) fixed in place with microscrews.

### Harvesting and preparation of the specimens

Under general anesthesia, three sheep each were killed at 4, 8, 12, or 16 weeks after grafting by using an overdose (40 mL) of Narcoren (Merial, Germany). In addition, 6–7 mL of heparin sodium (Aventis, Germany) was injected intravenously to prevent blood coagulation after death. The grafts were exposed and harvested en bloc. Specimens consisted of the (1) bone graft, (2) overlying soft tissue, and (3) the underlying mandibular cortical bone. The formalin-fixed specimens were decalcified with 10% CH_2_O_2_ for 4–5 weeks depending on the volume of the specimen. After dehydration the specimens were embedded in paraffin wax. For analysis, 10 - μm sections were cut from the paraffin-embedded tissue samples. (Microtome Leica RM 2255).

Immunohistochemical staining was performed according to local standard protocols. The specimens were deparaffinized in xylene and rehydrated in a graded alcohol series. After washing with 0.05 M Tris-buffered saline (TBS) and treatment of the specimens with CH_3_OH and H_2_O_2_, to block endogenous peroxidase and to avoid false-positive results, the samples were treated with ammonium chloride and Triton X (Schwarz/Mann Biotech, USA) in TBS to increase the permeability of the cell walls. Incubation with 5% bovine serum antibody (PAA Laboratories, UK) was followed by treatment with a primary antibody at a pretested concentration (1:200). After incubation with the primary antibody for 24 h at 4°C, incubation with the secondary antibody for 60 min, and washing with TBS, streptavidin biotinylated horseradish peroxidase complex (Amersham Biosciences, Germany) was added. For color development, a diaminobenzidine solution (Sigma-Aldrich, USA) was used. Color development was monitored and standardized for all stainings with the specific antibody.

**Figure 2:**
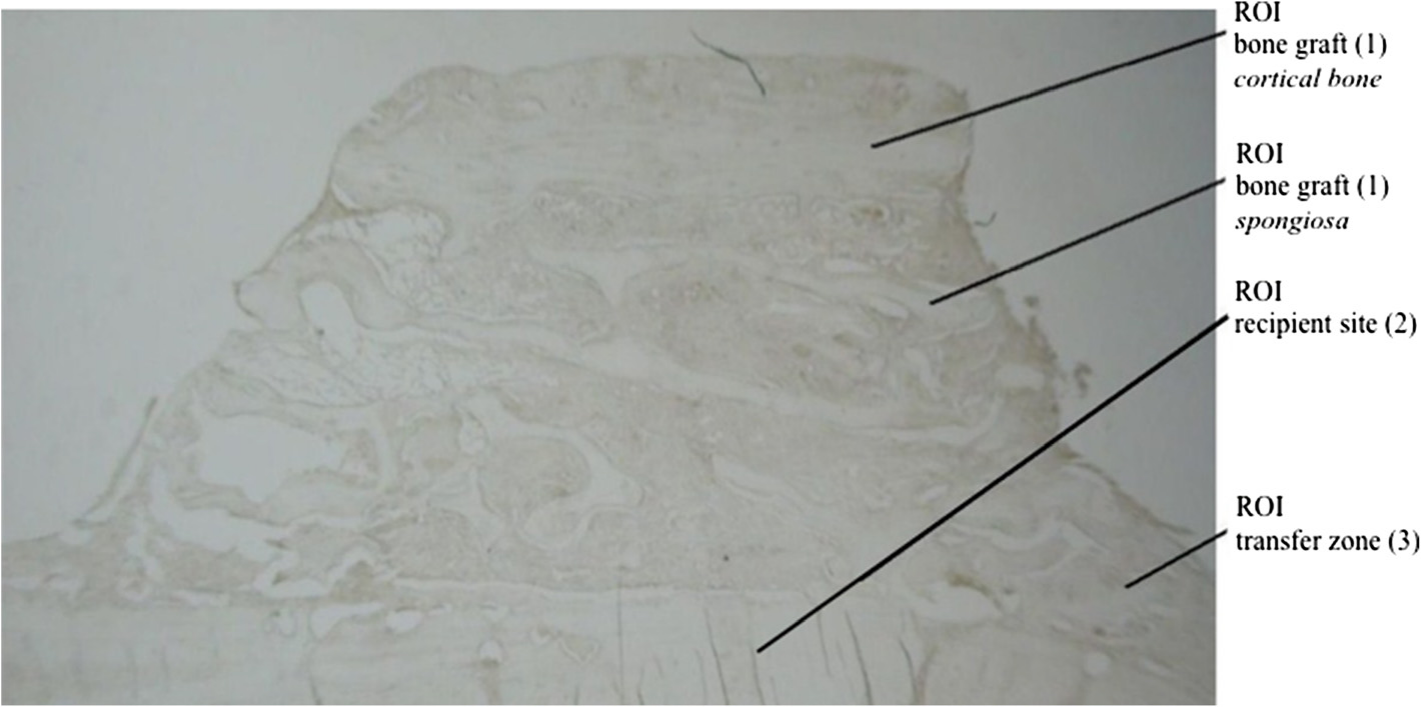
Photomicrograph of an unstained specimen illustrating the different regions of interest (ROI). (magnification, 16×).

The specimens were photographed under magnification (Leitz DM RBE/RD [Leica], Camedia C-4040Zoom [Olympus]) and divided into certain regions of interest (ROI) - (1) bone graft, (2) recipient site, and (3) transfer zone - that were used for the analysis. Figure 2 shows the different ROIs within an unstained histological photomicrograph.

**Figure 3:**
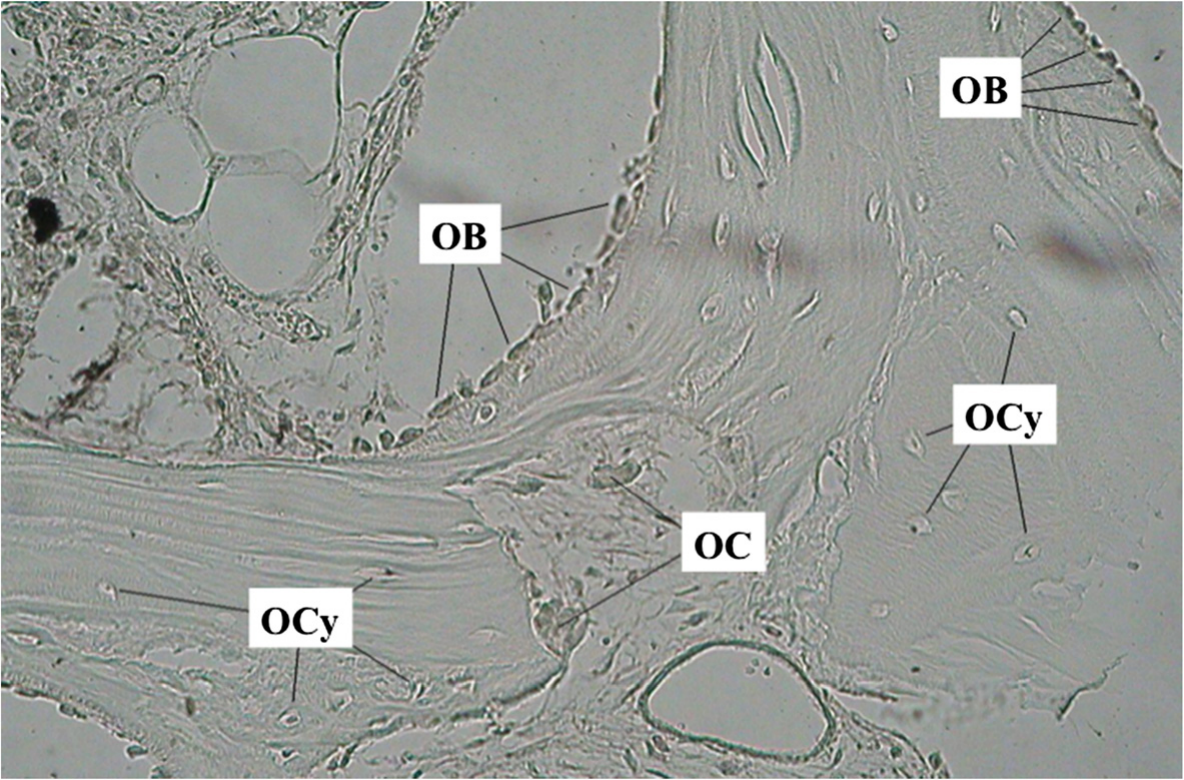
**Photomicrograph analyzing the different types of cells within the ROI “bone graft”.** OCy, osteocyte; OC, osteoclast; OB, osteoblast. (mangnification, 200×).

All photomicrographs were closely studied and (1) osteocytes (OCy), (2) osteoblasts (OB), and (3) osteoclasts (OC) were identified as shown in Figure 3.

To illustrate the revascularization processes vessels in connective tissue (CT) and bone were analyzed in the ROI “bone graft”. Moreover, the amount of CT was evaluated. High-power fields with 200× magnification were used for analysis. The density of vessels in CT and bone was assessed using a three-level scale: 0 = none, 1 = weak, 2 = moderate, and 3 = strong/dense. The amount of CT was analyzed according to the scale mentioned above.

The positivity (POS) of the immunostaining was assessed using a three-level scale in which negative to 0 indicated 0–5% positive cells, 1 indicated >5–20% positive cells, 2 indicated >20–50% positive cells, and 3 indicated >50–100% positive cells. Intensity (I) was graded as follows: 0 = none, + = weak, ++ = moderate, and +++ = strong/intense. For all mean values, the immunoreactive score was calculated as follows: IRS = [(I_Investigator1_ + I_Investigator2_)/2] × [(POS_Investigator1_ + POS_Investigator2_)/2]; minimum value = 0; maximum value = 9 [28].

All specimens were evaluated by two independent and specially trained investigators (S.K. and J.S.) who were blinded at the time of analysis.

### Data analysis

Because of the small sample size, specimens from 4 and 8 weeks after augmentation were classified as measurement 1 (M1) samples; all specimens from 12 and 16 weeks after the operation were classified as measurement (M2) samples.

All data were analyzed using SPSS for Mac (version 17.0; SPSS, Chicago, IL, USA). Means and SDs were calculated, and tests of significance were performed. For normally distributed values, the t-test was performed. For values non-normally distributed values the Mann–Whitney-test was used. Before the t-test, the Levene´s test was used to assess the equality of the samples. The statistical significance was adapted to multiple testing. Statistical significance was defined as α = 0.05.

## Results

In experiment 2, infection occured in two animals during measurement 1. These were not included in the analysis.

### Vessels in CT and bone

**Figure 4:**
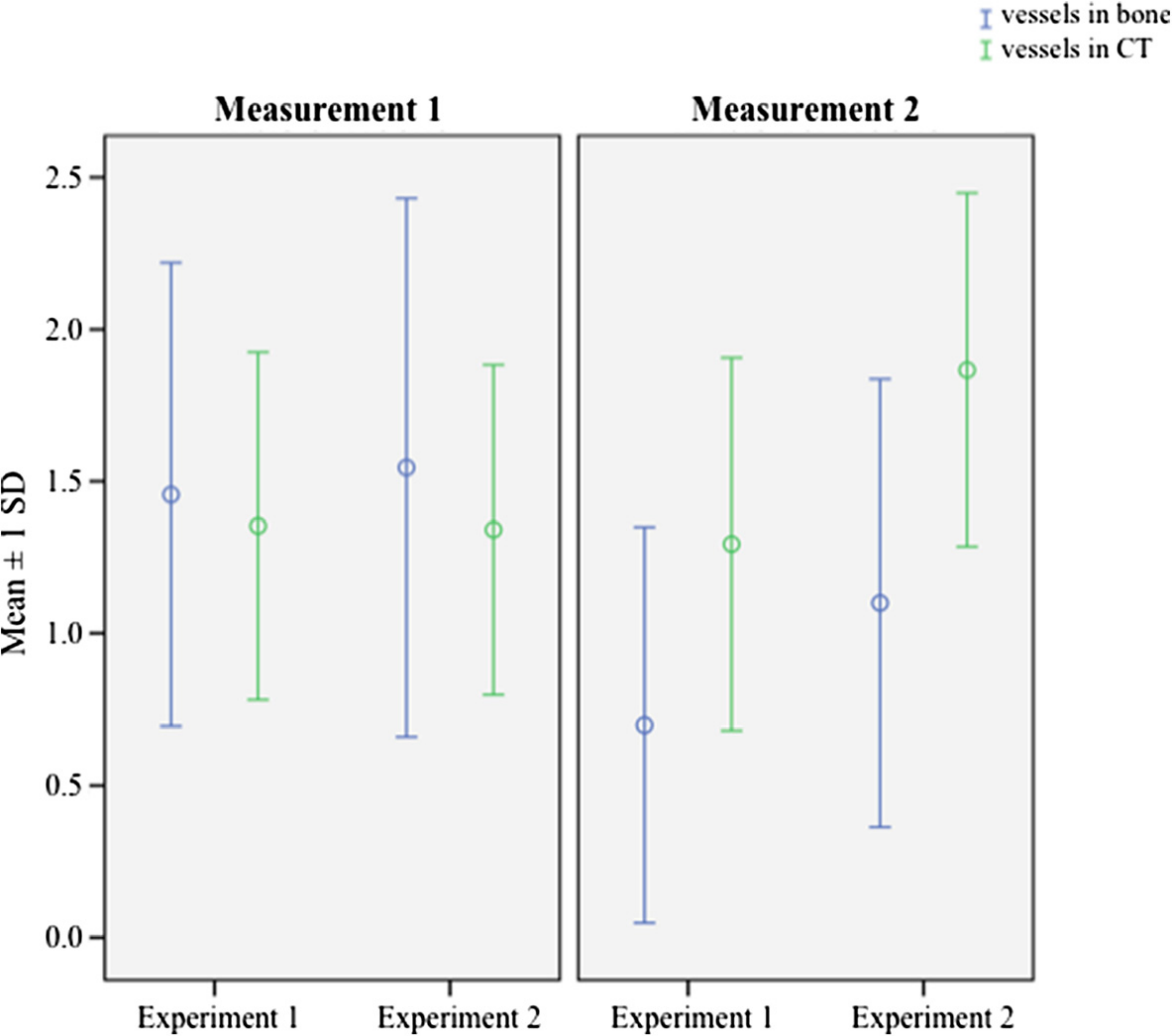
Graphic illustration of the three-level score for evaluating the amount of vessels within bone and connective tissue (CT) according to the different experiments and measurements.

Overall, the amount of vessels in the two experimental groups was at a similar level during measurement 1. Table 1 summarizes the findings after 4–8 weeks of graft healing. Considering the two measurements over time, the amount of vessels in experimental group 1 decreased, whereas it stayed almost constant in experiment 2 with GBR and DBBM as described in Table 2. 4–8 weeks after transplantation of the bone graft more vessels could be found in CT than in bone. At measurement 2, after a postoperative period of 12–16 weeks, this situation changed and more vessels were detected in bone. Figure 4 shows the amount of vessels in CT and bone according to the different measurements over time and experimental groups. In statistical analysis the difference between the amounts of vessels in CT in experiment 1 at both measurements was highly significant (1.46 ± 0.76 vs. 0.69 ± 0.65; *p* < 0.001). The amount of vessels in bone in experiment 2 at both measurements was increased (1.75 ± 0.60 vs. 1.33 ± 0.53; *p* < 0.05). After 12–16 weeks of incorporation of the bone grafts, vessels were statistically significantly increased in CT and bone (0.69 ± 0.65 vs. 1.10 ± 0.74; 1.75 ± 0.60 vs. 1.40 ± 0.65; all *p* < 0.05).

**Table 1:** Mean values and standard deviations of the three-level score evaluating the amount of vessels in connective tissue (CT) and bone at measurement 1

	**Experiment**	**N**^**a**^	**Mean**	**Standard-deviation**
Number of vessels in bone	1	93	1.457	0.758
	2	23	1.565	0.870
Number of vessels in connective tissue	1	94	1.360	0.571
	2	23	1.332	0.534

^a^N = number of specimens.

**Table 2:** Mean values and standard deviations of the three-level score evaluating the amount of vessels in connective tissue (CT) and bone at measurement 2

	**Experiment**	**N**^**a**^	**Mean**	**Standard-deviation**
Number of vessels in bone	1	64	0.688	0.652
	2	15	1.100	0.737
Number of vessels in connective tissue	1	81	1.413	0.646
	2	18	1.750	0.600

^a^N = number of specimens.

### Amount of CT

**Figure 5:**
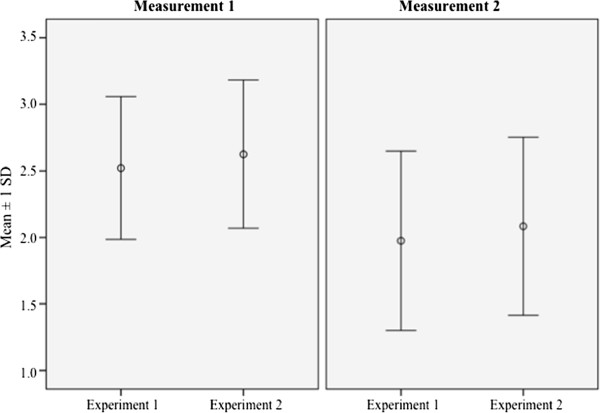
Graphic illustration of the three-level score for evaluating the amount of connective tissue (CT) according to the different experiments and measurements.

The combination of GBR and the use of DBBM in bone transplants as in experiment 2 showed, at both measurements, a lower rate of CT within the bone graft compared with experiment 1 (simple onlay bone graft). This difference was not statistically significant at any measurement (2.63 ± 0.56 vs. 2.52 ± 0.54; 2.08 ± 0.67 vs. 1.98 ± 0.67; all *p* > 0.05). In both experimental groups, a decreasing amount of CT over time was observed, as shown in Figure 5. The descriptive statistics can be found in Table 3. The increased amount of CT in simple onlay bone grafts at measurement 1 compared with measurement 2 is statistically highly significant (2.52 ± 0.54 vs. 1.98 ± 0.67; *p* < 0.001). The decreased expression of CT over time in experiment 2 also shows statistical significance in data analysis (2.62 ± 0.56 vs. 2.08 ± 0.67; *p* < 0.05).

**Table 3:** Mean values and standard deviations of the three-level score evaluating the amount of connective tissue (CT) according to the different experiments and measurements

**Measurement**		**N**^**a**^	**Mean**	**Standard-deviation**
1	Experiment 1	95	2.521	0.536
	Experiment 2	24	2.625	0.556
2	Experiment 1	79	1.976	0.674
	Experiment 2	18	2.083	0.669

^a^N = number of specimens.

### Immunohistochemical staining for vWF

**Figure 6:**
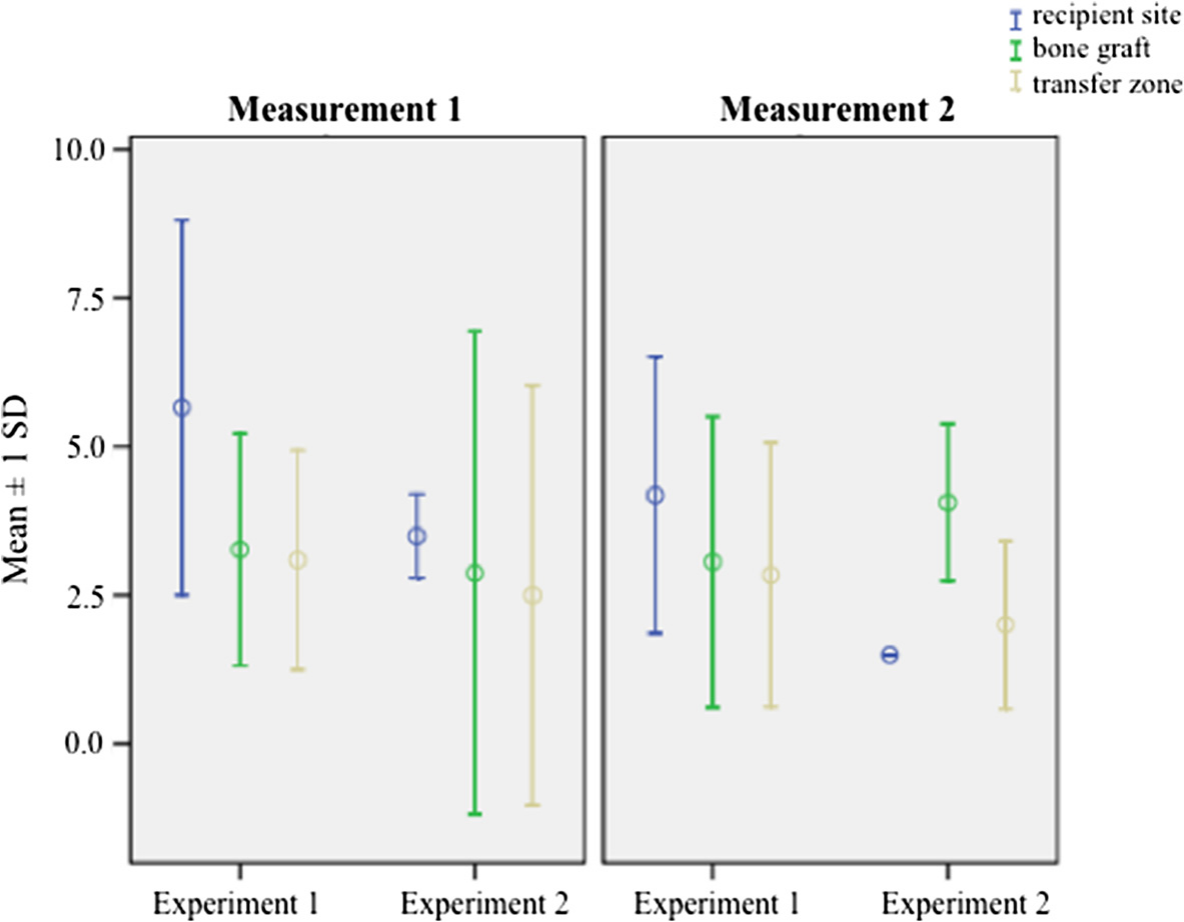
Graphic illustration of the immunoreactive score (IRS) in the ROIs “recipient site”, “bone graft” and “transfer zone” after immunohistochemical staining against vWF according to the different experiments and measurements.

Figure 6 shows the expression of vWF for experiments 1 and 2. An increased expression could be observed within the ROI “recipient site” (4.32 ± 2.71 vs. 3.06 ± 2.11; *p* = 0.79). Taking the two measurements over time into consideration, both experimental groups showed a statistically significantly higher expression at M1 (3.96 ± 2.46 vs. 2.84 ± 2.14; *p* < 0.05). Experiment 1 shows a higher overall expression of vWF than experimental group 2 (3.68 ± 2.44 vs. 2.74 ± 1.96; *p* = 0.22).

## Discussion

The unpredictable resorption and structural loss of integrity of simple onlay bone grafts in alveolar ridge augmentation procedures is still a limiting factor in their clinical use [8-12,29-31]. Resorption occurs especially within the first year after transplantation [32]. For periods from 6 to 18 months after the procedure, a loss in strength from up to 33% in cortical bone grafts is reported [33].

De Marco and colleagues conducted a study in rats that compared bone grafts covered with an expanded polytetrafluoroethylene (e-PTFE) membrane and simple autogenous onlay grafts [30]. Histomorphologic analysis was done 3, 7, 14, and 21 days after surgery. Revascularization occurred in both experimental groups, although the authors were able to show that vascular sprouts entered the graft from the host side as well as from the surrounding CT in the experimental group with the e-PTFE membrane covered graft.

Adeyemo et al. were able to show the clinical advantages of the use of DBBM in combination with GBR [4]. They illustrated that recipient bed perforation did not show any advantage over nonperforation; however, they reported that retention of the overlying periosteum resulted in better volume maintenance of the bone graft [3].

The present study compared autogenous bone grafts—simple onlay grafts to the mandible and onlay bone grafts combined with GBR and DBBM—with special attention to revascularization, by means of histomorphological and immunohistochemical analysis.

The use of GBR in mandibular augmentation procedures results in reduced resorption, less atrophy and better incorporation of the bone graft [7,10-12,34,35]. The concept of GTR/GBR is based on the assumption that cells migrate into a defect at different rates: epithelial and connective tissue both migrate at a faster rate, whereas bone-forming cells migrate at a slower rate. GTR/GBR adopts this concept and regulates the proliferation of different cell types within the graft by means of membranes, preventing fast migrating cells from entering the defect area and allowing the slower migrating bone-forming cells to multiply [10-12]. DBBMs, such as Bio-Oss®, are used in the clinic to support bone grafts in cases of insufficient bone volume at the host side. Especially the combination of autogenous bone and DBBM results in accelerated *de novo* bone formation in osseous defects, as shown by Thorwarth et al. in a porcine model [36]. The authors assume this accelerating effect on bone formation to be due to the osteoconductive properties of cellular elements transplanted with autogenous bone in combination with DBBM.

Other study groups such as Galindo-Moreno and colleagues were able to show the advantages of DBBM in combination with autogenous bone grafts in maxillary sinus augmentation procedures in a clinical study model [37].

In this study, the healing period was extended up to 16 weeks after surgery compared with previous studies [29,30].

Considering the amount of vessels in CT and bone, this study was able to show that the amount of vessels was at a constant level over time in experiment 2, in which DBBM and GBR were used to improve the incorporation of the graft. This is incongruent with result of De Marco and associates, who described a more intensive and more extensive revascularization in the experimental group in which onlay bone grafts alone were used [30]. Moreover, the amount of vessels in CT was higher than in bone at measurement 1 and this ratio was reversed after a healing period of 12–16 weeks. De Marco et al. showed that angiogenesis primarily occurred from CT to the graft. Considering that we extended the healing period up to 16 weeks, whereas De Marco and colleagues focused on the first 3 weeks after augmentation, these findings seem to be approximately equivalent.

We found a statistically significant decrease in the amount of CT within the bone graft over time in both experimental groups. Two different reasons for the reduced amount of CT in the different groups seem possible. In experiment 1, which showed a higher rate of resorption and atrophy in the clinical setting, CT and bone both decreased simultaneously because of inferior healing conditions [3,4,21]. For experimental group 2, an initial competition between regeneration of bone and the in-growing CT at measurement 1 seems a reasonable postulate. During the surveillance period of 16 postoperative weeks, revascularization processes in the bone graft seem to account for the decreased amount of CT, which is being replaced by newly formed bone.

In the current study, vWF as a marker protein of the endothelium was used for the immunohistochemical study of angiogenesis. VWF (factor VIII-related antigen), synthesized by endothelial cells and megakaryocvytes is a glycoprotein, that mediates platelet adhesion and stabilizes factor VIII at sites of vascular injury [38]. Moreover vWF can also be detected in thrombocytes. Although it is commonly used as an immunohistochemical marker for endothelial cells, stainings for other specific endothelial markers such as CD31, CD34, and Fli-1 should be performed in future studies to verify the current results [39]. Nevertheless, the increased expression in the ROI “recipient site” emphasizes the pattern of revascularization from the host site to the bone graft.

The biocompatibility of DBBMs such as Bio-Oss® has been shown in previous studies [40]. However, the two cases of infection in this study occured in experiment 2 with the use of a DBBM cover. DBBM is a xenograft material and foreign to the body. Although it is deproteinized, others report about low levels of osteoinductive or immunogenic proteins within the spongiosa granules [41,42]. In this context, an intense and prolonged immune response seems to be possible. As DBBMs are resorbed slowly, the potential of infection should not be underestimated. These effects have to be studied in long-term follow-up clinical trials.

Taking the results of this study and previous studies into consideration, it seems that the use of a CM in combination with DBBM in autogenous bone grafts provides benefits for bone tissue regeneration in terms of revascularization, reduced resorption and less atrophy of the graft compared with simple onlay bone grafts [3,4,21]. The fact that angiogenesis precedes osteogenesis and that newly formed bone is always found in close relation with newly formed vessels, indicates the close connection of angiogenesis and osteogenesis [43]. If this is true only because of the protective effects of GBR or if, and how much, the potential angiogenetic properties of DBBM also promote this effect has to be investigated in further studies.

## Conclusion

This study shows more intensive and extensive revascularization in onlay autogenous iliac grafts for lateral alveolar ridge augmentation with the use of GBR and DBBM compared with simple onlay bone grafts.

## Competing interests

The authors declare no conflict of interests.

## Authors´ contributions

SK, JS, WB, ACK and TR conceived of the study and participated in its design and coordination. OR made substantial contributions to conception and design of the manuscript as well as statistical analysis. SK, JS, and OR have been involved in drafting the manuscript. WB, ACK and TR were involved in revising the manuscript. All authors read and approved the final manuscript.
